# Premature aging induced by radiation exhibits pro-atherosclerotic effects mediated by epigenetic activation of CD44 expression

**DOI:** 10.1111/acel.12253

**Published:** 2014-07-25

**Authors:** Donna Lowe, Kenneth Raj

**Affiliations:** Biological Effects Department, Centre for Radiation, Chemical and Environmental Hazards, Public Health EnglandChilton, Didcot, OX11 0RQ, UK

**Keywords:** CD44, endothelium, irradiation, inflammation, monocyte adhesion, senescence

## Abstract

Age is undoubtedly a major risk factor for heart disease. However, the reason for this is not entirely clear. In the course of our investigation into the mechanism of radiation-induced cardiovascular disease, we made several unexpected findings that inform us on this question. We observed that human coronary endothelial cells, while being able to initiate repair of radiation-induced DNA damage, often fail to complete the repair and become senescent. Such radiation-induced cellular aging occurs through a mutation-independent route. Endothelial cells that aged naturally through replication or as a result of radiation exhibited indistinguishable characteristics. The promoter regions of the *CD44* gene in aging endothelial cells become demethylated, and the proteins are highly expressed on the cell surface, making the cells adhesive for monocytes. Adhesion is a cardinal feature that recruits monocytes to the endothelium, allowing them to infiltrate the vessel wall and initiate atherosclerosis. The epigenetic activation of CD44 expression is particularly significant as it causes persistent elevated CD44 protein expression, making senescent endothelial cells chronically adhesive. In addition to understanding why cardiovascular disease increases with age, these observations provide insights into the puzzling association between radiation and cardiovascular disease and highlight the need to consider premature aging as an additional risk of radiation to human health.

## Introduction

The potency of ionizing radiation lies in its ability to damage DNA. Misrepair of such damage introduces mutations, some of which may be carcinogenic. However, cancer is not the only radiation-associated pathology. Exposure to ionizing radiation is also strongly associated with development of cardiovascular disease (CVD) (Adams *et al*., 2003; Mone *et al*., 2004; Little, 2010; Shimizu *et al*., 2010), which is an age-related pathology. This puzzling association was most impressively revealed in studies which demonstrated that women who underwent radiotherapy for left breast cancer, receiving higher doses of radiation to the heart, acquired significantly higher risk of developing CVD compared with women who had right breast radiotherapy, and even higher than women who did not receive radiotherapy (Darby *et al*., 2003; Taylor *et al*., 2008, 2009). Intriguingly, these women developed CVD years after radiotherapy. It is not known whether radiation-induced CVD is distinct or similar to age-related CVD, for which the most common cause is the emergence of atherosclerotic plaques in the wall of the coronary artery (Libby, 2006a). The rupture of plaques triggers clotting within the artery, causing myocardial infarction. While several different hypotheses have been proposed to explain how atherosclerotic plaques form (Ross *et al*., 1977; Williams & Tabas, 1995; Libby, 2006b), there is general agreement that insudation of low-density lipoprotein (LDL) and recruitment of monocytes to the vessel intima are two pivotal events. LDL in the intima stimulates endothelial cells (ECs) to express surface proteins, which retard the rolling of monocytes on the endothelial cell surface, the first step in the transit of monocytes from the lumen into the intima, where they transform into macrophages and take up insudated cholesterol. This further transforms them into foam cells. This process eventually results in the formation of a plaque consisting of oxidized cholesterol, cell debris and foam cells enveloped by smooth muscle cells (Libby, 2002, 2006b).

The combined knowledge of biological effects of radiation (which has hitherto been confined to induction of mutation) and atherogenesis (which is an age-related disease unrelated to mutations) does not immediately proffer an obvious explanation of how radiation can impact on plaque formation. This dearth of conceptual common ground suggests that not all biological effects of radiation have been fully elucidated or appreciated. Taking this view, we set out to investigate whether ionizing radiation affects steps that lead to atherosclerotic plaque formation.

We considered the essential early atherogenic step of monocyte entry into the blood vessel wall. For this to occur, ECs have to be able to retard the flow of monocytes in the blood stream. This is elicited by increased adhesiveness of the ECs for monocyte. We tested whether adhesiveness of ECs that line the coronary artery is affected by radiation. A radiation dose of 10 Gy was used in our investigations as it is within the total dose range that the heart receives in breast cancer radiotherapy. In keeping with the delayed effect of radiation on CVD, biological effects of radiation were examined at time points of weeks after irradiation. Should pro-atherogenic effects be present after such times, it is more likely that they would be relevant in the eventual development of atherosclerotic plaques.

## Results

### Adhesion of monocytes on endothelial cell layer after X-irradiation

To ensure sufficient supply of primary cells for the study, primary endothelial cells of human coronary artery (European Cell Culture Collection) were immortalized with retroviruses bearing the *est2* gene, a yeast homologue of the human TERT protein. Although all experiments were carried out with the immortalized endothelial cells, the majority of the experiments were also repeated with the use of nonimmortalized primary ECs, with additional repeats with a second EC donor for some. These results are referred to in the text and shown as figures in the Supporting Information section.

**Figure 1:**
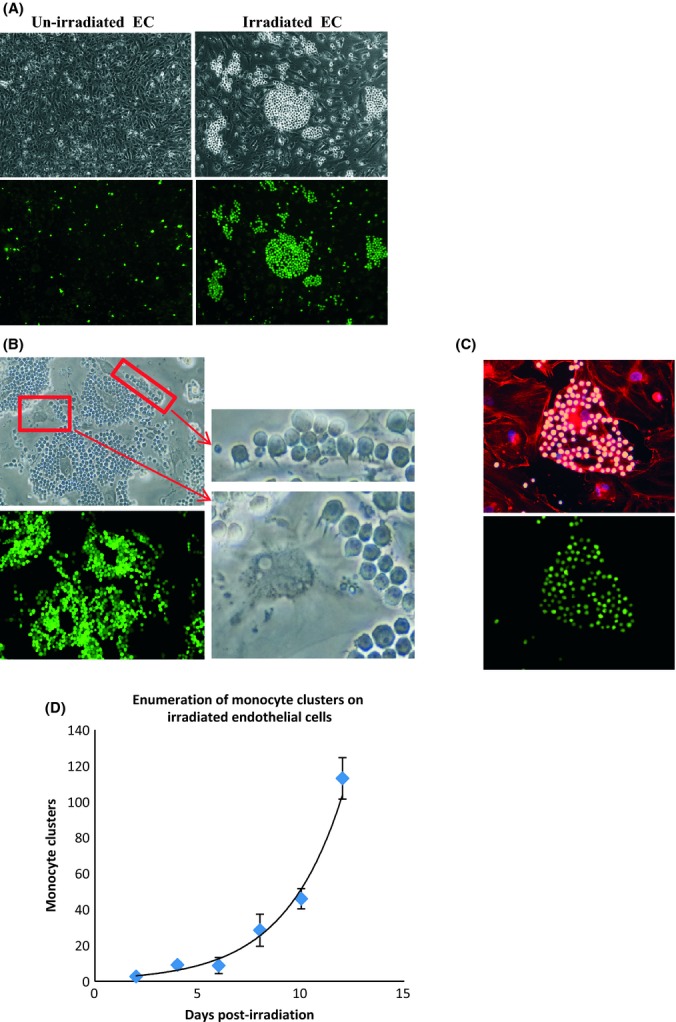
Enhanced adhesion of monocytes on irradiated endothelial cells (A) Upper panels are phase contrast pictures of un-irradiated or 1 week postirradiated (10 Gy) ECs with monocytes. The lower panels are the same fields with only cell tracker green-labelled monocytes visible. A 10× objective was used. (B) Green monocyte clusters in the lower panel identify monocytes in the upper left panel attached to 3 weeks postirradiated ECs. These two images were photographed with a 20× objective. Magnification with 40× objective of two regions within the upper panel images highlights the projections that monocytes used to anchor on to irradiated ECs. (C) Upper panel showing a field of 3 weeks postirradiated (10 Gy) ECs, of which only one was bound by monocytes. Cell nuclei (purple), microtubules (red) and monocytes (yellow). Lower panel of the same field showing only monocytes (green). (D) Number of monocyte clusters were counted from five regions of a coverslip (113 mm^2^), with three coverslips scored for each time point. The results were plotted in function time post-10 Gy irradiation of ECs. These observations were made repeatedly over ten times.

ECs grown on glass coverslips were irradiated (10 Gy), and after a week, HL-60 monocytes (human promyelocytic leukaemia cells) were deposited onto them, and unattached cells rinsed off after incubation. While individual monocytes adhered sporadically at low numbers to un-irradiated ECs, clusters of monocytes formed on the irradiated endothelial monolayer (Fig. 1A). Higher magnification revealed that monocytes attached to ECs through fine projections from their membrane (Fig. 1B). This specificity was accompanied by a very high level of selectivity as seen in Fig. 1C where monocytes bound to an individual EC, but not to others surrounding it.

To quantify this effect, we counted monocyte clusters (where at least 10 monocytes around a single EC were considered a cluster) and found that ECs began to increase their adhesiveness approximately 1 week after irradiation (Fig. 1D). Beyond 15 days, the monocyte clusters were too numerous and began to merge, precluding accurate scoring. The percentage of adhesive ECs within the irradiated population can be determined by dividing the number of monocyte clusters by the total number of ECs within a field (Fig. S1, Supporting Information). Such an analysis carried out 12 days postirradiation revealed that 8–10% of irradiated ECs acquired increased adhesiveness (Table 1). This, however, is an underestimation as the number of monocyte clusters increased in time beyond accurate numeration. In spite of this, an 8–10% frequency of effect is at least a thousand times greater than that expected, were EC adhesion be caused by mutation of a single gene (0.01%) induced by a similar X-ray dose (Ellender *et al*., 2005). As such, radiation-induced adhesiveness of ECs is mediated by a mechanism that is independent of mutation.

**Table 1:** Quantitation of adhesive ECs 12 days post-10 Gy irradiation

	Percentage of adhesive endothelial cells		
	Disc 1	Disc 2	Disc 3
Field 1	8.65	8.29	8.21
Field 2	10.44	7.27	7.21
Field 3	9.63	9.05	8.33
Field 4	11.11	7.09	8.41
Field 5	10.55	9.4	11.61
Average	10.07	8.22	8.75

Monocyte clusters on irradiated ECs grown on glass cover slips (discs) were counted in five separate fields and the number divided by the total number of ECs in the field to obtain percentage of adhesive ECs.

### Characterization of adhesive postirradiated ECs

**Figure 2:**
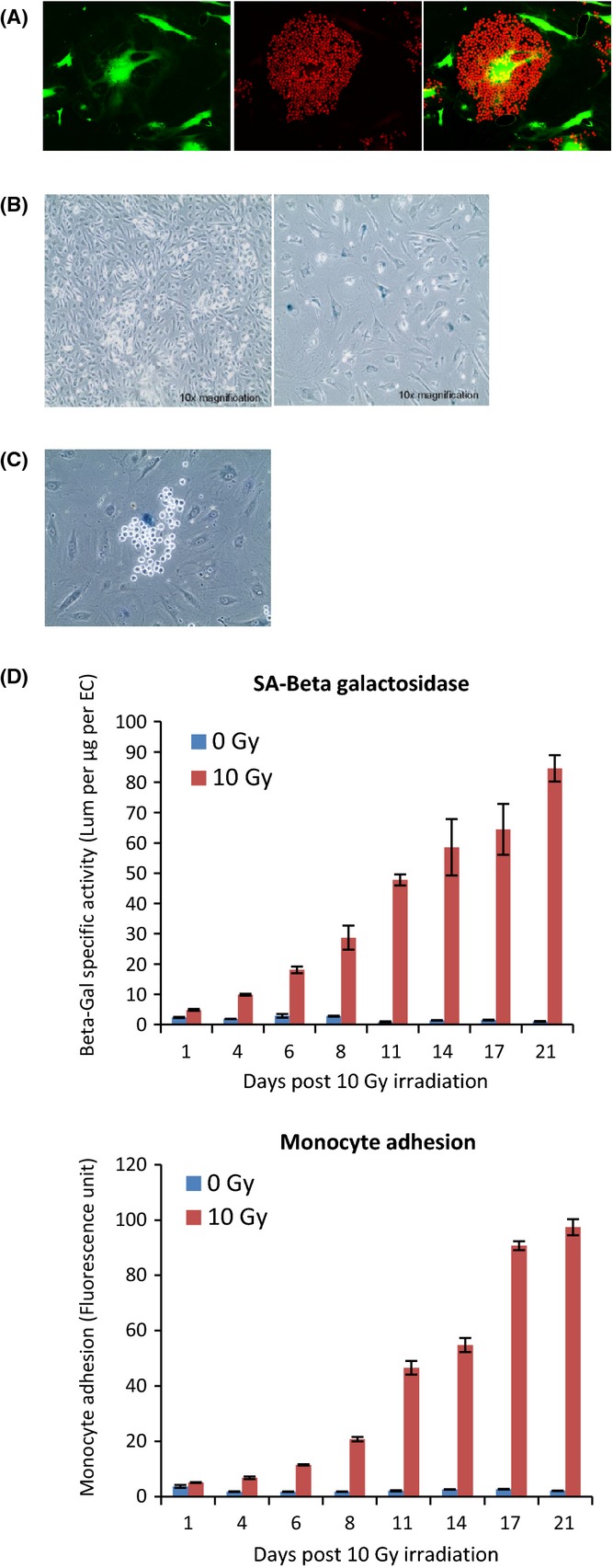
Adhesive ECs are viable but senescent (A) Three weeks postirradiated (10 Gy) ECs were first stained with calcein AM dye to reveal viable cells (left), followed by incubation with monocytes labelled with Cell Tracker Red (middle and right). Experiment was repeated twice. (B) Comparative images of un-irradiated (left panel) and 3 weeks postirradiated (10 Gy, right panel) ECs that were stained for SA beta-Gal (blue). Both images were taken with a 10× objective. Experiment was repeated at least four times. (C) Irradiated ECs subjected to monocyte adhesion assay followed by staining for SA beta-Gal (blue). Experiment was repeated more than five times. (D) Quantitative SA beta-Gal assay (top panel) and quantitative monocyte adhesion assay (lower panel) of ECs irradiated (10 Gy) or not, in function of time postirradiation. Data were from triplicate samples within an experiment. Experiment was repeated four times.

It was apparent that not all irradiated ECs were rendered adhesive by radiation. To ascertain the selective criteria that mark an irradiated EC for adhesion by monocytes, we first determined whether monocytes were adhering to irradiated ECs that were dead. Calcein AM, which becomes fluorescent only in live cells, was added to the media of irradiated (10 Gy) ECs prior to carrying out monocyte adhesion assay. It can be seen in Fig. 2A that monocytes adhered to living postirradiated ECs. Next, we tested whether cells with unrepaired damaged DNA were the targets of monocyte adhesion. We stained irradiated ECs with antibodies against γ-H2AX, a marker of damaged DNA. At early time points after irradiation, cells were stained with numerous γ-H2AX foci. Although the number of foci decreased in time, they did not entirely vanish even after 2 weeks (Fig. S2), indicating that while DNA repair was initiated, some damaged DNA remained unrepaired. The fact that all 2 weeks postirradiated ECs possessed damaged DNA and yet only a subset amongst them were bound by monocytes (Fig. S3), suggests that damaged DNA *per se* is not the criterion for monocyte attachment.

We noticed that irradiated ECs were larger than un-irradiated ones (Fig. S4), and within the irradiated population, a subset of cells were very much larger and reminiscent of senescent cells. Staining of 2 weeks postirradiated ECs for senescence-associated beta-galactosidase (SA beta-Gal) that colours lysosomes of senescent cells blue revealed that the giant irradiated ECs were indeed senescent (Fig. 2B). SA beta-Gal staining after monocyte adhesion assay revealed that senescent ECs were selectively bound by monocytes (Figs 2C and S5). Quantitative measurement of SA beta-Gal activity of EC populations revealed that within a period of 3 weeks, SA beta-Gal activity increased steadily from day 6 postirradiation in irradiated cell populations, and this was mirrored by a similar and parallel rise in adhesion of monocytes as determined by quantitative monocyte adhesion assay (Fig. 2D). Collectively, these results suggest that monocytes selectively adhered to irradiated ECs that have become senescent.

**Figure 3:**
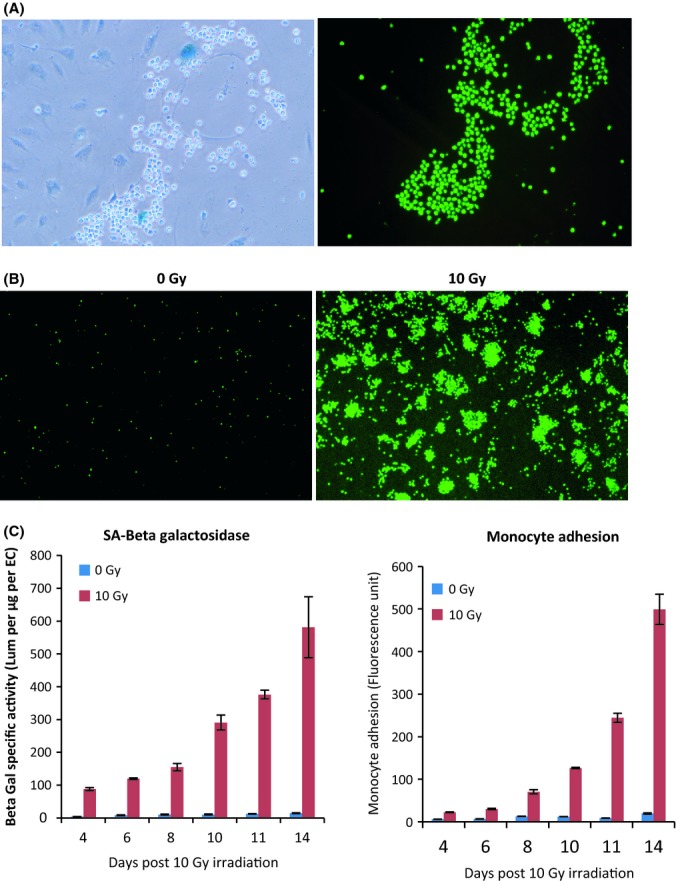
Monocytes adhere to replicative senescent ECs (A) Un-irradiated and nontransduced ECs approaching replicative senescence were subjected to monocyte adhesion assay and subsequently stained for SA beta-Gal to reveal senescent cells (blue). The left panel shows phase contrast image, and the right shows the fluorescence image of the same region. The reduced number of monocytes in the left panel is an unavoidable consequence of the manipulations during the staining procedure. Images were captured with 40× objective. Experiment was repeated three times. (B) Monocyte adhesion assay carried out with Cell Tracker Green-labelled monocytes on nonimmortalized ECs 14 days postirradiation. Images were captured with 10× objective. Experiment was repeated twice with ECs from different donors. (C) Left panel: quantitative SA beta-Gal assay of nonimmortalized ECs in function of time postirradiation. Right panel: quantitative monocyte adhesion assay of nonimmortalized ECs in function of time postirradiation. Experiment repeated twice.

Next, we tested whether this phenomenon is specific to radiation-induced senescent ECs or if it is also a feature of ECs that senesce naturally through replication (replicative senescence). Un-irradiated primary (nontelomerase transduced) ECs were passaged in culture until senescent cells (identified by their increased size) began to appear. At this point, monocyte adhesion assays were carried out, followed by SA beta-Gal staining. It can be seen in Fig. 3A that monocytes adhered readily and specifically to nonirradiated replicative senescent giant ECs (large cells stained blue). When irradiated, nonimmortalized ECs also exhibited monocyte adhesion, which increased in parallel with SA beta-Gal activity and in function of time (Fig. 3B,C). These results show that senescent ECs that arose, either by irradiation or naturally through exhaustive replication, acquired augmented adhesiveness to monocytes.

### Characterization of proteins involved in monocyte adhesion on irradiated endothelial cells

**Figure 4:**
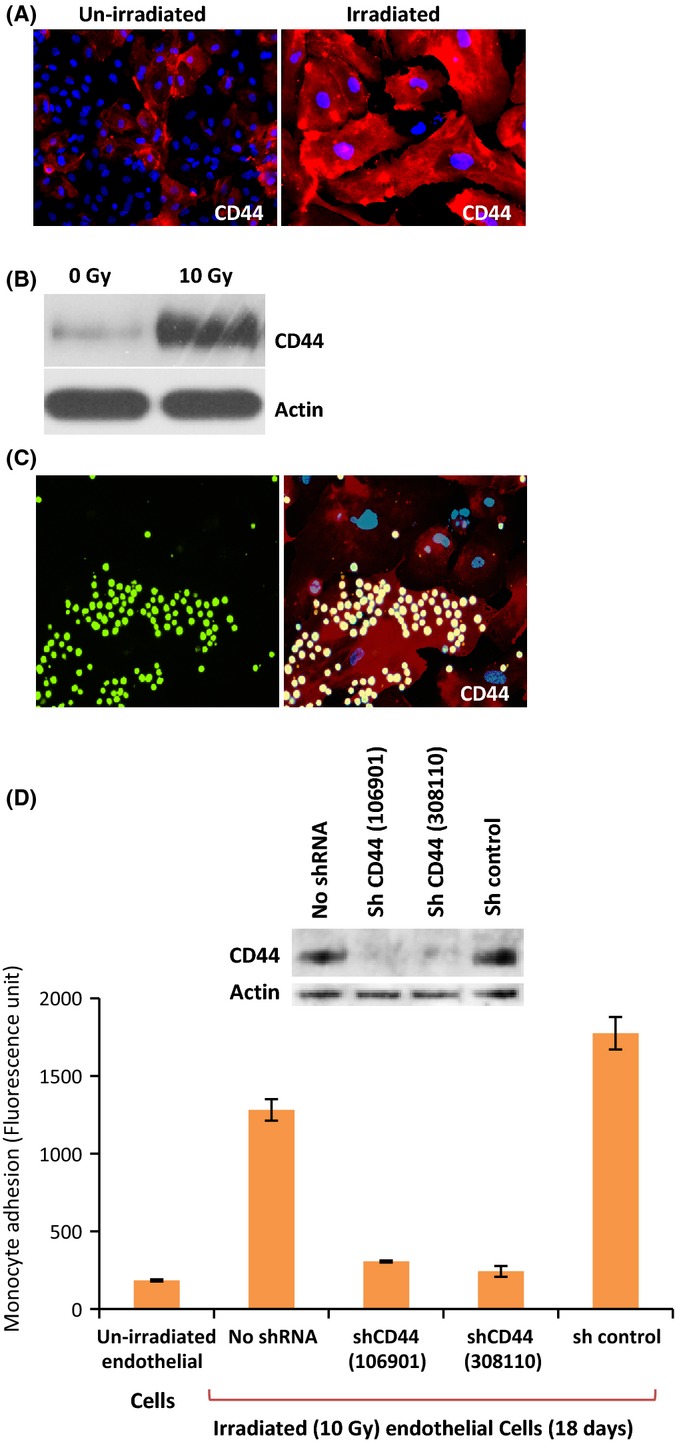
Induction of CD44 expression by radiation (A) Unirradiated and 19 days postirradiated (10 Gy) ECs were stained with anti-CD44 (red). Both images were captured with a 20× objective. (B) Western blot analyses of cell extracts from un-irradiated and 2 weeks postirradiated (10 Gy) ECs using antibodies against CD44, and Actin. (C) Monocyte (green) adhesion assay on 3 weeks postirradiated ECs followed by immunofluorescence staining of CD44 (red). (D) Top panel shows western blot of lysates from irradiated ECs infected with lentivirus expressing shRNA against CD44 [ShCD44 (106901) and ShCD44 (308110)] or control lentivirus bearing scrambled sequence. Western blot was probed with anti-CD44. Lower panel shows the results of quantitative monocyte adhesion assay on ECs described above. Data were from triplicate samples within an experiment. Experiment was repeated twice.

To determine what makes senescent ECs particularly adhesive for monocytes, we analysed the levels of several adhesive molecules and found CD44 to be of particular interest. We observed that while un-irradiated ECs exhibited limited and sporadic CD44 staining, irradiated ones expressed very high levels of this protein (Fig. 4A), with some cells expressing much higher levels than others (Fig. S6). The increase of CD44 by irradiation was also demonstrated by Western blotting (Fig. 4B). Notably, CD44 was also selectively expressed in un-irradiated replicative senescent primary (nontelomerase transduced) ECs (Fig. S7), supporting the possible importance of CD44 in EC adhesiveness, as reported (Mun & Boo, 2010). Consistent with this, we observed monocyte clusters to form on irradiated ECs that expressed much higher levels of CD44 (Fig. 4C). To be certain that CD44 is indeed the radiation-induced molecule responsible for monocyte attachment, irradiated ECs were infected with lentiviruses expressing short hairpin RNAs against CD44. Western blot in Fig. 4D shows that the CD44 protein level was indeed reduced by lenti-shCD44 but not in shControl cells. Quantitative adhesion assays revealed that irradiated ECs with reduced CD44 did not exhibit augmented adhesion while control irradiated cells did, confirming that CD44 is indeed essential for radiation-induced EC adhesiveness.

### Analyses of *CD44* gene expression

**Figure 5:**
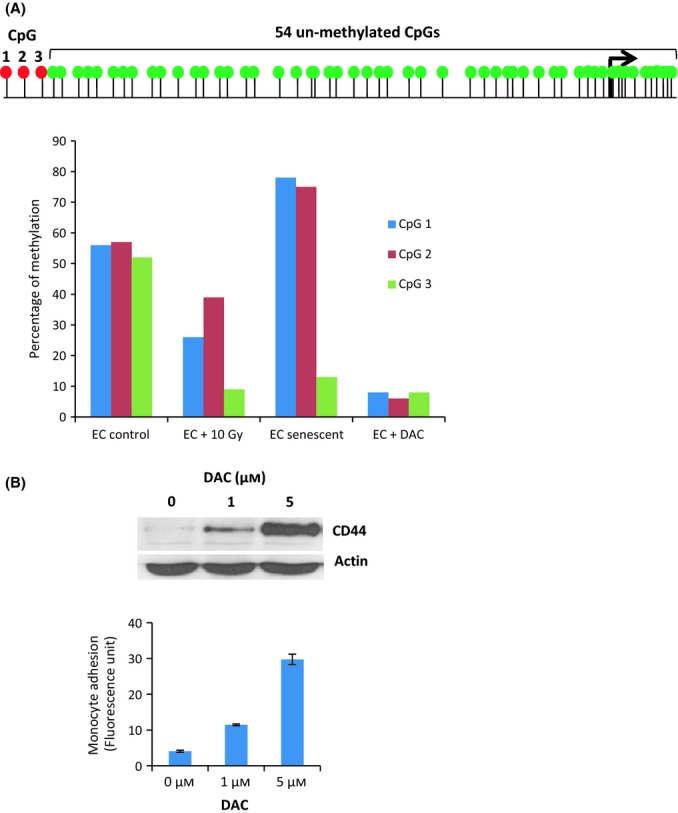
Demethylation of CD44 promoter (A) Upper panel: Depiction (not to scale) of relative positions of CpGs to each other and the transcription start site (arrow). Green dots are CpGs that are not methylated, while red dots are CpGs that are methylated in ECs. Numbers 1,2 and 3 signifies CpGs whose methylation states in untreated, irradiated, senescent or 5 μm DAC-treated ECs, are shown in the bar chart in the lower panel. Each bar represents percentage of methylation from bi-sulphite sequencing of 48 clones of the CD44 promoter (EC control = 4 biological replicates, EC irradiated = 3 biological replicates, EC senescent = 2 biological replicates, from two different EC donors, EC + DAC = 2 biological replicates) (B) Upper panel: CD44 and actin immune-blots of protein lysates of ECs 10 days post-treatment with different amounts of DAC. Lower panel: Quantitative adhesion assay of these cells after 10 days treatment with DAC.

To determine what triggers increased CD44 expression by irradiation, we quantified CD44 transcripts by quantitative PCR and observed a fourfold increase (Fig. S8). It was previously reported that CD44 promoter is activated by deazacytidine (DAC) (Kagara *et al*., 2012), prompting us to test whether irradiation might increase CD44 expression through demethylation of this promoter. Sequencing the CD44 promoter after bisulphite treatment revealed that in un-irradiated ECs, of the 57 CpGs analysed, all were unmethylated but for three CpGs at position −638 (CpG1), −627 (CpG2) and −607 (CpG3) upstream of the translational start. Upon irradiation, methylation at these three CpGs were reduced, with CpG3 exhibiting the greatest diminution (Fig. 5A). Importantly, CpG3 has been demonstrated to be crucial for activating the CD44 promoter when demethylated (Kagara *et al*., 2012). CpG3 was the sole CpG demethylated in un-irradiated nonimmortalized ECs that underwent replicative senescence, suggesting a lack of requirement for demethylation of CpG1 and CpG2 in enhancing CD44 expression, as previously reported (Kagara *et al*., 2012). When CpG demethylation was elicited in ECs by DAC treatment (Fig. 5A), CD44 protein expression was augmented, with a dose-dependent rise in adhesiveness of the ECs for monocytes (Fig. 5B), confirming that the CD44 promoter can be activated to increase CD44 expression and EC adhesiveness by DNA demethylation.

### Characterization of CD44’s participation in monocyte adhesion

**Figure 6:**
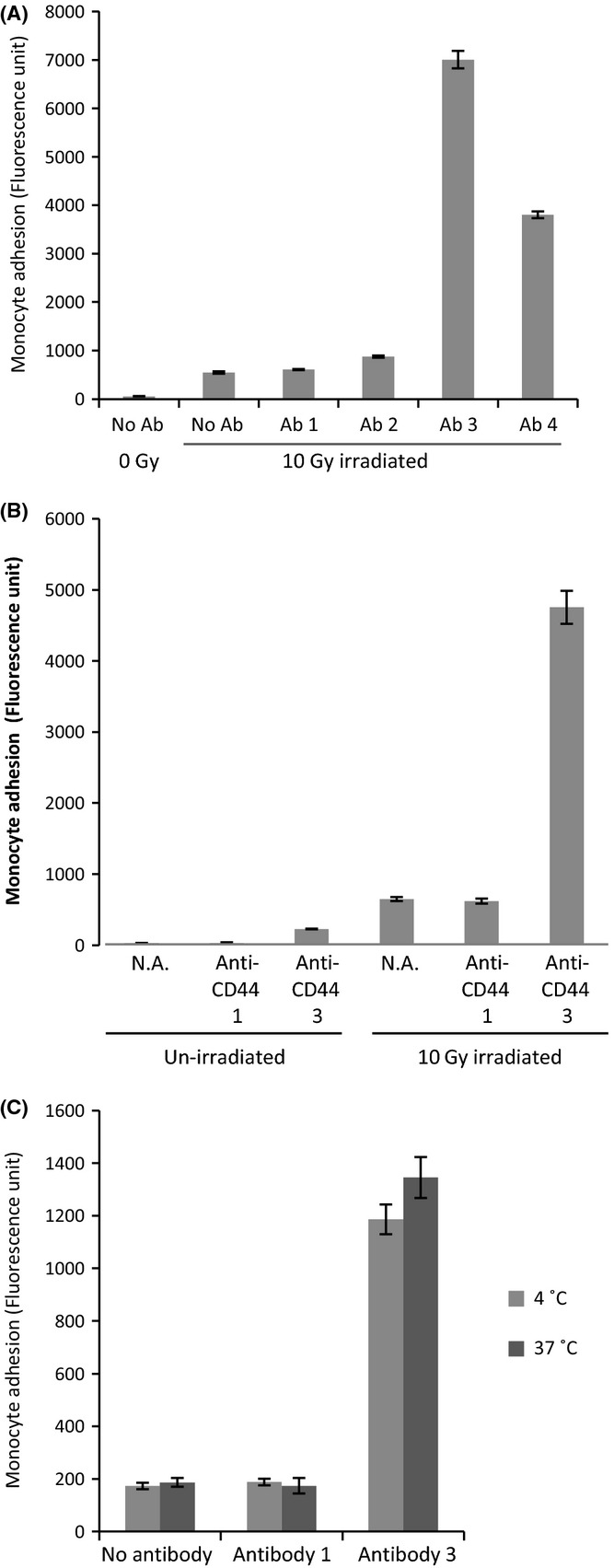
Anti-CD44 increases monocyte adhesion on irradiated ECs (A) Two weeks postirradiated (10 Gy) ECs were preincubated with no antibodies or different antibodies against CD44 followed by adhesion assay, alongside un-irradiated ECs. Antibody 1, Cell Signalling 3578; antibody 2, Calbiochem 217594 that blocks interaction with hyaluronan; antibody 3, Pharmingen 550392 which recognizes epitope 1 of CD44 or antibody 4, Ancell 352-820, which recognizes epitope 2 of CD44 (6). Data were from triplicate samples within an experiment. This experiment was repeated four times (B) Unirradiated EC or 2 weeks postirradiated (10 Gy) ECs were preincubated with no antibodies (N.A.), CD44 antibody 1 (Cell Signalling 3578) or CD44 antibody 3 (Pharmingen 550392) followed by monocyte adhesion assay. This experiment was repeated three times. (C) Two weeks postirradiated ECs were preincubated with no antibodies, CD44 antibody 1 or CD44 antibody 3 as described above but with one set of preincubation at 4 °C and the other at 37 °C, followed by quantitative monocyte adhesion assay. Data were from triplicate samples within an experiment. This experiment was repeated twice.

To characterize how CD44 participates in monocyte adhesion, ECs were preincubated separately with a panel of different CD44 antibodies, prior to monocyte adhesion assay. Figure 6A shows that antibodies directed to epitope 1 (antibody 3 – BD Pharmingen 550392, Franklin Lakes, NJ, USA) and epitope 2 (antibody 4 – Ancell 352-820) of the CD44 protein increased adhesion of monocytes to irradiated ECs. The adhesion enhancement was so substantial that it was obvious even by visual examination alone, as shown in Fig. S9. CD44 antibody 1 and antibody 2, which do not recognize these CD44 conformations, did not enhance adhesion (Fig. 6A). Importantly, antibody 2 (Calbiochem 217594, Billerica, MA, USA), which has been demonstrated to block CD44-hyaluronan interaction, did not inhibit radiation-induced adhesiveness of ECs. Likewise, antibody 4, which enhanced radiation-induced adhesiveness, is also a CD44–hyaluronan blocking antibody (Liao *et al*., 1995; Bourguignon *et al*., 2006), showing that the adhesion of monocytes to irradiated ECs is independent of hyaluronan. Preincubation of nonirradiated ECs with CD44 antibody 3 also enhanced adhesion by monocytes, but the magnitude of enhancement was marginal and very much lower than that of irradiated ECs (Fig. 6B). This is consistent with the fact that CD44 levels in the un-irradiated ECs were very low, and demonstrating that adhesion enhancement elicited by CD44 antibodies is mediated by CD44 proteins. The possibility that Fc receptors on HL-60 were responsible for adhesion was tested and excluded as preincubation of irradiated ECs and HL-60 with Fc receptor inhibitor (Affymetrix eBioscience 16-9161, San Diego, CA, USA) prior to adhesion assay did not prevent monocyte adhesion on irradiated ECs (Fig. S10). To determine whether adhesion-enhancing CD44 antibodies imposed their effect indirectly via triggering of intracellular signalling cascade, the experiment above was repeated with the antibody preincubation step at 37 or 4 °C prior to adhesion assay. The results in Fig. 6C show that temperature did not affect adhesiveness of irradiated ECs, suggesting that enhancement of monocyte adhesion instigated by CD44 antibodies is likely through direct self-association of CD44 molecules on the cell surface.

## Discussion

The most compelling reports that highlight the association between radiation and CVD are those demonstrating that women who underwent radiotherapy for left breast cancer acquired higher risks of developing CVD (Darby *et al*., 2003; Roychoudhuri *et al*., 2007). The cumulative dose to the hearts of these subjects could be as high as 17 Gy (Taylor *et al*., 2008, 2009). It is intriguing that CVD, which is characteristically a nonmutation linked ailment, can be induced by radiation, whose pathological effect has hitherto been ascribed to its mutagenic property. This phenomenon cannot be readily explained from current understanding of radiation effects on cells. Hence, we began by testing the effects of radiation on one of the earliest pro-atherosclerotic events: adhesion of monocyte on endothelial cells.

The high-level selectivity of monocyte attachment on irradiated ECs is obvious in numerous figures in this report. Although irradiated ECs become larger, this selectivity is not owed to greater surface area of the target cell as the image in Fig. S11 shows that cells with even greater surface area adjacent to the EC bound by monocytes were not targeted. The selectivity was accompanied by specificity, which is evident from the fact that monocytes were not resting passively on irradiated ECs, but projected sections of their membranes to anchor onto the ECs. Concerns that the high level of adhesion induction triggered by irradiation could be an anomaly due to a peculiarity of HL-60 cells or to the particular batch of ECs were eliminated when primary monocytes isolated from peripheral blood and ECs derived from another donor also exhibited such strong radiation-induced adhesion (Fig. S12). Although increased monocyte adhesion on irradiated ECs has been previously reported (Colden-Stanfield *et al*., 1994; Hallahan *et al*., 1996; Khaled *et al*., 2012), the appearance of monocyte clusters around individual ECs has never been described. These monocyte clusters allowed quantitation of radiation effects on individual ECs for the first time. This revealed two important features. The first is a delay in the manifestation of EC adhesion after irradiation. Interestingly, this delay is associated with the delayed onset of senescence of the irradiated EC population. Secondly, quantification of monocyte clusters around individual ECs revealed that at least 8–10% of the irradiated population of ECs became adhesive 12 days postirradiation. This is about a thousand times greater than expected if this was a mutation-driven mechanism (Ellender *et al*., 2005), demonstrating a departure from the standard model of radiation-induced mutation-dependent phenotypic changes. This, to our knowledge, is the first quantitative demonstration of a nonmutagenic route by which radiation can induce a potentially pathological cellular change. This change is achieved through the induction of cellular senescence, which is a consequence of continued presence of damaged DNA (Suzuki *et al*., 2001; Muthna *et al*., 2010). However, it is puzzling that not all irradiated ECs became senescent after irradiation in spite of the fact that they all had long-lasting DNA damage. We are unaware of any obvious reason for this, but considering that nonsynchronous EC populations were irradiated in the experiments above, it is possible that radiation-induced senescence is dependent on the position of the cell within the cell cycle at the time of irradiation. Alternatively, the location of damage on the DNA may be crucial, and damage to telomeric regions might be those that lead to senescence (Hewitt *et al*., 2012). Although the role of senescent cells in pathology, especially with regard to cancer, is gaining acceptance, their role in atherosclerosis is less appreciated in spite of the fact that giant ECs have long been observed in aged blood vessels and on atherosclerotic plaques (Repin *et al*., 1984; Tokunaga *et al*., 1989) and demonstrated to be senescent and to increase in number with age (Vasile *et al*., 2001; Minamino *et al*., 2002). Our experiments demonstrate that senescent ECs, by being highly adhesive for monocytes, exhibit at least one feature that is undoubtedly pro-atherosclerotic. This supports the notion that senescent ECs are not a consequence but a contributor of atherogenesis (Minamino *et al*., 2002; Brandes *et al*., 2005; Erusalimsky & Kurz, 2005; Erusalimsky, 2009). Importantly, whether senescence was induced by radiation or through exhaustive replication, demethylation at position −607 (CpG3) of the CD44 promoter increased expression of CD44, causing these cells to become adhesive for monocytes. Collectively, these features demonstrate that ionizing radiation can induce a pro-atherosclerotic effect through premature aging of ECs. This induction of premature aging, to our knowledge, is the first demonstration of the potential pathological effect of radiation-induced cellular senescence. These observations also provide a long-awaited perspective into why risk of cardiovascular disease increases with age, a link that is well recognized but hitherto without any molecular explanation.

The involvement of CD44 as the adhesive molecule on senescent ECs is surprising as it has thus far been largely associated with cancer stem cells (Jaggupilli & Elkord, 2012; Geng *et al*., 2013). However, as CD44 is expressed in multiple isoforms (Naor *et al*., 1997), its role in cancer cells and senescent cells may be mediated by different isoforms. Although it is not known which CD44 ligands are involved in the adhesion of monocytes to irradiated cells, hyaluronan can be ruled out as two antibodies that block CD44–hyaluronan interaction did not inhibit monocyte adhesion. Instead one of the antibodies even enhanced adhesion. Although antibody-induced enhancement of adhesion appears counter-intuitive, it is not unprecedented. Oostendorp *et al*. (Oostendorp *et al*., 1996; Bendall *et al*., 1997) showed after testing a panel of nineteen anti-CD44 antibodies, that while none of them blocked hematopoietic cells from adhering to bone marrow stroma, nine of them actually enhanced binding. The majority of those that did were antibodies directed to epitope 1 of CD44, which is also the epitope recognized by the CD44 antibody (antibody 3) that exhibited the greatest enhancement of monocyte adhesion. It is unlikely that the enhancement was elicited indirectly via intracellular signalling as antibody preincubation at 4 °C did not impede the enhancement. It is notable that monocytes bound preferentially to ECs with higher CD44 expression and that preincubation with adhesion-enhancing CD44 antibodies resulted in virtually all irradiated ECs (but not un-irradiated ones) becoming adhesive (Fig. S9). As CD44 expression is augmented to different levels in irradiated ECs (Figs 4C and S6), these antibodies might act by aggregating CD44 proteins on the cell surface, endowing adhesiveness to irradiated ECs whose augmented levels of CD44 were otherwise below the threshold for adhesion to occur without assistance. This postulation deserves greater in-depth study because factors (such as antibodies) that affect CD44 association can have a profound effect on the adhesiveness of senescent ECs. Identification of molecules that prevent CD44 association may provide a realistic and highly accessible means by which adhesion of monocytes to senescent ECs may be mitigated, and atherosclerotic plaque development stalled or prevented.

An intuitive next step in studying the role of CD44 is to test its importance in atherosclerosis *in vivo*. This, fortuitously, has already been demonstrated by Cuff *et al*. (Cuff *et al*., 2001), who showed that atherosclerotic lesions in ApoE −/− mice were reduced by 50–70% when the mice were also null for CD44. Furthermore, CD44 expression was also shown to be increased in function of the severity of atheromatous plaques (Krettek *et al*., 2004). Although these excellent reports highlighted the importance of CD44, it remained puzzling how CD44 contributed to plaque formation, a question that is now addressed by the observations above. An important feature of atherogenesis is chronic inflammation, a condition thought to be *sine qua non* for the development of atherosclerotic plaques (Libby, 2006b; Libby *et al*., 2010). In this regard, senescent ECs, by being long-lived and chronically adhesive (via CD44) for monocytes, fulfil the required criteria. It is significant that the role of CD44 in inflammation has been described (Johnson *et al*., 2000; Pure & Cuff, 2001) and demonstrated to be important for radiation-induced atherosclerosis in ApoE−/− mice (Hoving *et al*., 2008). It is evident that permanent expression of CD44 on senescent ECs is required to sustain inflammation. As such, the demethylation of CD44 promoter in senescent ECs is of particular importance. Demethylation at CpG3 position was previously demonstrated to correlate very strongly with persistently high expression of CD44 (Kagara *et al*., 2012). Hence, it is notable that while CpG1 and CpG2 methylation were altered differently between irradiated and replicative senescent ECs, CpG3 was consistently demethylated in both. Collectively, these results show that demethylation of CpG3, regardless of causative factor (radiation, aging or Dnmt inhibition by drugs), is sufficient to enhance CD44 expression and EC adhesiveness. The most important implication of this epigenetic activation of the CD44 promoter is that it allows long-term adhesiveness (hence inflammation) of senescent ECs, induced either through replicative senescence or irradiation and possibly other DNA-damaging agents.

Overall, the observations made in this study have (i) demonstrated that that radiation-induced DNA damage induces cellular changes that are akin to those acquired through natural aging (replicative senescence); (ii) provided a plausible molecular explanation for the long-standing puzzle of the association between ionizing radiation and cardiovascular disease and raising the question of whether other noncancer diseases associated with radiation, such as cataract, are also induced through premature aging of cells in the affected tissue; (iii) demonstrated that ionizing radiation’s long-term effect on cells is beyond that of DNA mutation; (iv) shown that radiation can induce a specific epigenetic change that produces a biological effect that is potentially pathogenic; (v) provided strong support for the notion that senescent cells are not merely a bystander outcome of aging but that they possess biological properties that are potentially detrimental; (vi) highlighted the fact that secondary factors, through interaction with CD44, can affect adhesiveness of endothelial cells; and (vii) demonstrated the need to consider premature aging (in addition to cancers) in the risk assessment of radiation effects.

We are mindful that monocyte attachment is not the only process required for the formation of atherosclerotic plaques and that aging and radiation may also affect other cellular processes involved in atherogenesis, as has been demonstrated in ApoE−/− mice (Stewart *et al*., 2010; Seemann *et al*., 2012; Stewart, 2012). Hence, it is prudent to take a measured view of the effects of aging and radiation on monocyte attachment and consider it as one of several events that contribute to the eventual development of atherosclerotic plaques.

## Experimental procedures

### Primary endothelial cells from human coronary artery

ECs from human coronary artery were purchased from European Cell Culture Collection (HCAECs Cat. No: 300-05) and transduced with retroviruses bearing the *est2* gene, a yeast homologue of the human TERT protein. All experiments were carried out using cells between passage 6 and 22. All cells were mycoplasma free and cultured at 37 °C with 5% CO_2_ in a humidified incubator.

### Treatment of cells with deazacytidine

EC growth in DAC was titrated using 0, 0.2, 1.0, 5.0, 10.0, 15.0 and 20.0 μm DAC in the media. At concentrations of 1 and 5 μm, ECs were found to affect morphology minimally but still allowed the treated cells to proliferate. Cells were then grown in these concentrations with daily change of media with fresh DAC for 10 days before DNA was extracted and subjected to bi-sulphite modification and sequencing.

### Labelling of cells with Cell Tracker Green or Cell Tracker Red

Cell Tracker Green and Red (Invitrogen C7025 and C34552, Carlsbad, CA, USA) were used at 1 μm in RPMI devoid of FCS to label HL-60 cells and primary monocytes. Cells incubated with Cell Tracker for 1 h at 37 °C were resuspended in EC medium at one million cells mL^−1^ and used in adhesion assays described below.

### Labelling of cells with calcein AM

Calcein AM (Sigma C1359, St. Louis, MO, USA) was diluted a hundred times in EC medium, and 4 μL of this stock was added per millilitre of EC medium. After an hour of incubation in normal cell culture conditions, the medium was removed, and the cell monolayer rinsed once with Hanks buffered saline solution (HBSS) and replenished with EC medium.

### Senescence-associated beta-galactosidase assay

This assay was carried out using the Senescence β-Galactosidase Staining Kit 9860 from Cell Signaling Technology (Danvers, MA, USA) according to the manufacturer’s instructions.

### Monocyte adhesion assay

Fifty-thousand ECs were seeded into each well (of 24 well plate) containing a fibronectin-coated coverslip. Cells were cultured until confluent and irradiated. Five-hundred thousand Cell Tracker-labelled HL-60 monocytes in 0.5 mL EC medium were added to each well and incubated at 37 °C. After 1 h, the glass coverslip was picked up with forceps and rinsed by dipping ten times in HBSS, after which the edge of the coverslip was pressed on an absorbent paper to drain the HBSS. This procedure was carried out three times, after which the coverslip was fixed in formalin before being placed on Vectashield Hard Set mounting medium with 4′,6-diamidino-2-phenylindole (DAPI) (Vectorlabs H-1500). Agglomerations of ten or more monocytes on or around ECs were scored as clusters.

### Irradiation

Cells in their media were irradiated with 10 Gy X-ray at 250 kV and 13 mA at room temperature.

### Quantitative monocyte adhesion assay

Preparation of coverslips, seeding and growth conditions of ECs, monocyte incubation on the coverslips and the subsequent washings were identical to those described for monocyte adhesion assay above. After washing, the coverslip was placed in 0.5 mL of trypsin–EDTA for 5 min followed by 0.5 mL of soybean trypsin inhibitor to allow single-cell suspension to be prepared. Cells were pelleted and resuspended in 100 μL of HBSS and deposited into 96-well cluster plate with black sides. Monocyte fluorescence was measured using a Bio-TEK (Winooski, VT, USA) Synergy HT microplate reader, with kc4 software. To calculate fluorescence unit, fluorescence readings were divided by the number of ECs in respective 24 wells and the resulting values multiplied by either 100 or 1000 depending on the magnitude of the result.

### Generation of ECs expressing shRNA

Vectors carrying DNA-encoding shRNA against CD44 procured from Sigma (106901 and 308110) were transfected into 293TT cells with lentiviral packaging vectors (p8.91 and VSVG). Two days posttransfection, viruses in the media were harvested and mixed with 8 μg mL^−1^ polybrene and used to infect ECs. One day after infection, EC media were substituted with fresh media containing 1 μg mL^−1^ puromycin. Three days later, when all uninfected ECs control were killed, the surviving transduced ECs were used in the experiments described.

### Preincubation with antibodies or Fc receptor inhibitor

Medium was removed, and 0.3 mL of fresh medium containing 10 μg mL^−1^ of the appropriate antibody or Fc receptor inhibitor was added. Incubations at 37 or 4 °C were carried out for 30 min, after which the medium with antibodies was aspirated off and cells rinsed twice with EC medium before being subjected to adhesion assay described above. Antibodies used were anti-CD44; Cell Signalling 3578 (antibody 1), Calbiochem 217594 (antibody 2), BD Pharmingen 550392 (antibody 3) and Ancell 352-820 (antibody 4). Fc inhibitor (Affymetrix eBioscience Cat No: 16-9161) was used as recommended by the manufacturer.

### Preparation of total cell lysate

Cells were lysed in SDS lysis buffer (2% SDS, 100 mm Tris, pH 6.8), and lysates were centrifuged through a Qiashredder (Qiagen 79654, Valencia, CA, USA), and protein concentration was assayed using Pierce bicinchoninic acid assay (BCA protein assay 23224 and 23221) according to the manufacturer’s instructions. Cell lysates were stored at −70 °C until use.

### Western blotting

Fifty micrograms of total cell lysates were separated through a 4–10% SDS polyacrylamide gel at 120 V, after which the proteins were transferred to a PVDF membrane. The membrane was blocked in 5% low-fat dried milk in Tris-buffered saline–Tween-20 (TBS–Tween) for at least an hour followed by incubation with the appropriate antibodies for an hour.

### Immunofluorescence

Cells grown on coverslips were fixed in formalin (15 min), permeabilized in 0.1% Triton X-100 and blocked in 2% foetal calf serum (2 × 15 min). This was followed by incubation in primary antibody for one hour. After three washes in HBSS, appropriate Alexa 488 or Alexa 594-conjugated antibodies diluted 1:200 were added and incubated for 30 min followed by washing as described above. Coverslips were mounted on glass slides using Vectashield Hard Set mounting medium with DAPI. Antibodies used were as follows: anti-tubulin α (Santa Cruz SC-23948, Santa Cruz, TX, USA), anti-CD44 (BD Pharmingen 550392), anti-γH2AX (Abcam ab26350, Cambridge, UK) and anti-actin (Santa Cruz SC-1616).

### Bisulphite sequencing of CD44 promoter

DNA from cells were extracted with Qiamp minikit (Qiagen 51306) according to the manufacturer’s instructions, followed by bisulphite treatment according to the instructions that accompanied the EZ DNA Methylation Gold Kit (D5005) from Zymo Research (Irvine, CA, USA). The resulting DNA was subjected to amplification with three sets of overlapping primers which together amplify a region of 1004 bp, which stretches from position −846 upstream of the ATG to position 158 downstream of the ATG. The primers sequences are as follows: CD44-primer set 1 (Forward: *AGGAAGAGAG*AGTATGTGTGTGGAGA GAGGTGTTT, Reverse: *CAGTAATACGAC*AATTCAACCTTTAACCTCTCCTTTC), CD44-primer set 2 (Forward: *AGGAAGAGAG*AAAGGAGAGGTTAAAGGTTGAATTT, Reverse: *CAGTAATACGAC*AAAC ACACCCAAACAAAAAAAACTA), CD44-primer set 3 (Forward: *AGGAAGAGAGA*TAGTTTTTTTTGTTTGG GTGTGTT, Reverse: *CAGTAATACGAC*CAAACAACTCACTTAACTCCAATCC), CD44-primer set 4 (Forward: *AGGAAGAGAG*TTTGGGTTTTATAGGATGTTGGATA, Reverse: *CAGTAATACGAC*CCCTCACTCCCC ACTATAAACAC). The amplification conditions were as follows: 94 °C for 15 min followed by 45 cycles of 94 °C for 20 s, 60 °C for 30 s and 72 °C for 30 s. The amplification products were cloned into pGEM T Easy vector from Promega (A1360, Madison, WI, USA) according to the manufacturer’s instructions and transformed into JM109 bacteria. Forty-eight individual colonies of bacteria were picked for each condition and sent to Source Bioscience for sequencing. DNA sequences from irradiated and nonirradiated ECs were analysed and compared.

### Quantitative PCR of *CD44* gene expression

Gene expression level was determined by quantitative reverse transcription–PCR of total RNA extracted from ECs 14 days postirradiation with 10 Gy X-rays or un-irradiated control using the RNeasy Mini kit (Qiagen, Cat. 74104) followed by DNase digested. One microgram of RNA was reverse transcribed to cDNA using the RT^2^ First Strand Synthesis kit (Qiagen, Cat. 330401). qRT–PCR was performed using a Rotor Gene Q cycler (Qiagen) with cycling conditions of 10 min at 95 °C, then 40 cycles of 5 s at 95 °C and 45 s at 60 °C using PerfeCTa SYBR Green SuperMix (Quanta Biosciences. Cat. 95054, Gaithersburg, MD, USA), 300 mm of each primer and 1 μL cDNA in a 10 μL reaction. The primers used are as follows: CD44 forward CCCAGATGGAGAAAGCTCTG, reverse GTTGTTTGCTGCACAGATGG; HPRT forward TCAGGCAGTATAATCCAAAGATGGT, reverse AGTCTGGCTTATATCCAACACTTCG. Each sample and controls were run in triplicate and repeated twice. Fold change was calculated using 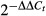 by normalization to a HPRT internal control then compared to the un-irradiated control sample.

## Supporting Information

**Fig. S1** Quantification of monocyte cluster frequency. **Fig. S2** Irradiated ECs initiated but failed to complete DNA repair. **Fig. S3** Adhesion of monocytes on irradiated ECs with damaged DNA. **Fig. S4** Increase in size of irradiated ECs. **Fig. S5** Adhesion of monocytes to senescent ECs. **Fig. S6** Irradiated ECs express varying levels of CD44 protein. **Fig. S7** Selective expression of CD44 in replicative senescent nonimmortalised ECs. **Fig. S8** Radiation induced transcription of CD44. **Fig. S9** Preincubation of irradiated EC with CD44 antibodies directed to epitope 1 increases monocyte adhesion. **Fig. S10** Inhibition of Fc receptor did not prevent adhesion of monocytes on irradiated ECs. **Fig. S11** Adhesion of monocytes on irradiated ECs is independent of cell surface area. **Fig. S12** Primary monocytes from peripheral blood and primary human coronary ECs from a different donor exhibited adhesiveness in response to radiation.
