# Supplementary material for: Premature aging induced by radiation exhibits pro-atherosclerotic effects mediated by epigenetic activation of CD44 expression
Source: Aging Cell. 2014 Jul 25;13(5):900–10. doi: 10.1111/acel.12253 (PMC4331742; doi:10.1111/acel.12253)
Supplement: Supplementary file 1 — Fig. S1 Quantification of monocyte cluster frequency. Fig. S2 Irradiated ECs initiated but failed to complete DNA repair. Fig. S3 Adhesion of monocytes on irradiated ECs with damaged DNA. Fig. S4 Increase in size of irradiated ECs. Fig. S5 Adhesion of monocytes to senescent ECs. Fig. S6 Irradiated ECs express varying levels of CD44 protein. Fig. S7 Selective expression of CD44 in replicative senescent nonimmortalised ECs. Fig. S8 Radiation induced transcription of CD44. Fig. S9 Preincubation of irradiated EC with CD44 antibodies directed to epitope 1 increases monocyte adhesion. Fig. S10 Inhibition of Fc receptor did not prevent adhesion of monocytes on irradiated ECs. Fig. S11 Adhesion of monocytes on irradiated ECs is independent of cell surface area. Fig. S12 Primary monocytes from peripheral blood and primary human coronary ECs from a different donor exhibited adhesiveness in response to radiation. [file acel0013-0900-sd1.pptx]

## Slide 1
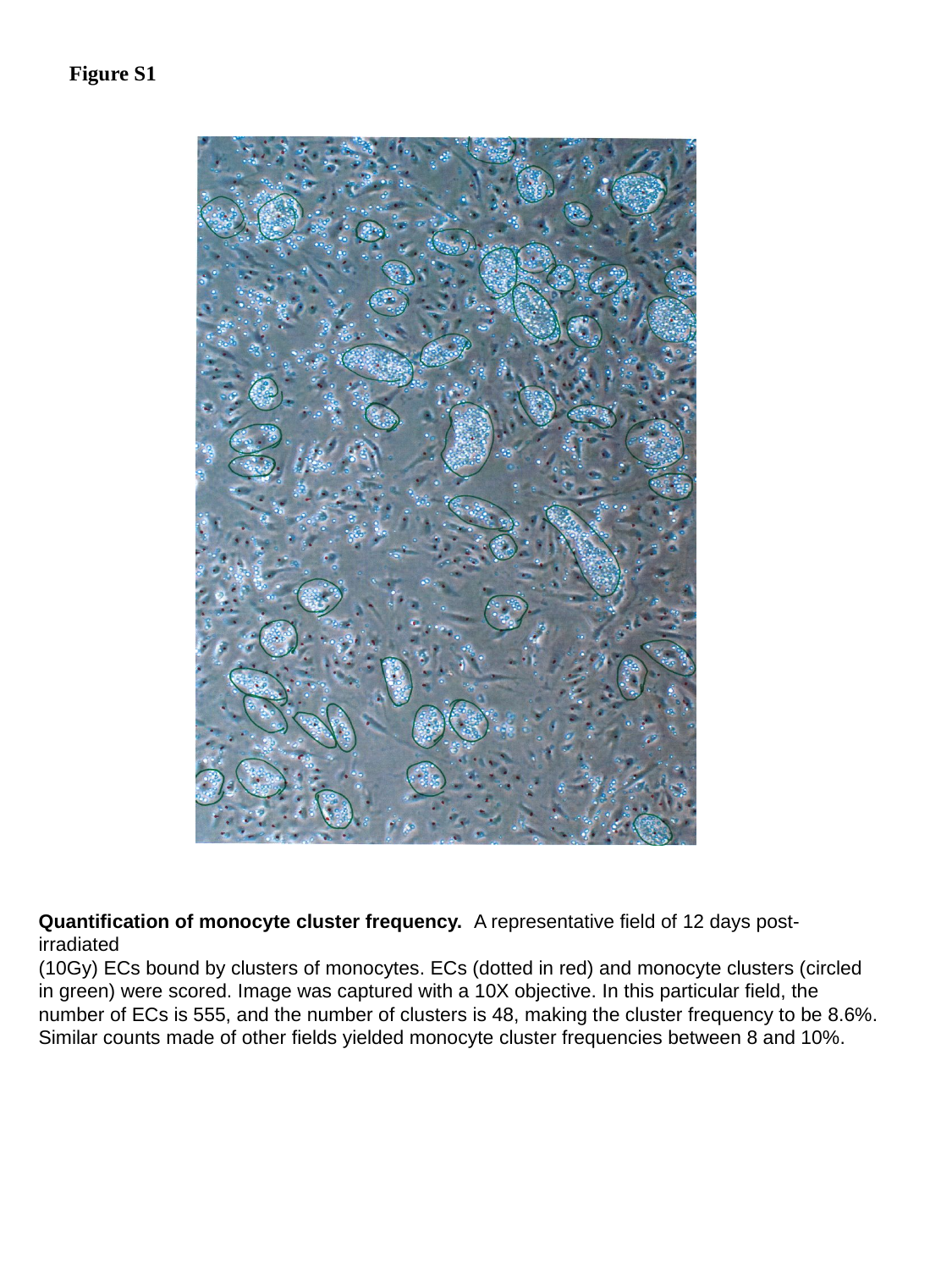

Figure S1
Quantification of monocyte cluster frequency. A representative field of 12 days post- irradiated
(10Gy) ECs bound by clusters of monocytes. ECs (dotted in red) and monocyte clusters (circled in green) were scored. Image was captured with a 10X objective. In this particular field, the number of ECs is 555, and the number of clusters is 48, making the cluster frequency to be 8.6%. Similar counts made of other fields yielded monocyte cluster frequencies between 8 and 10%.

## Slide 2
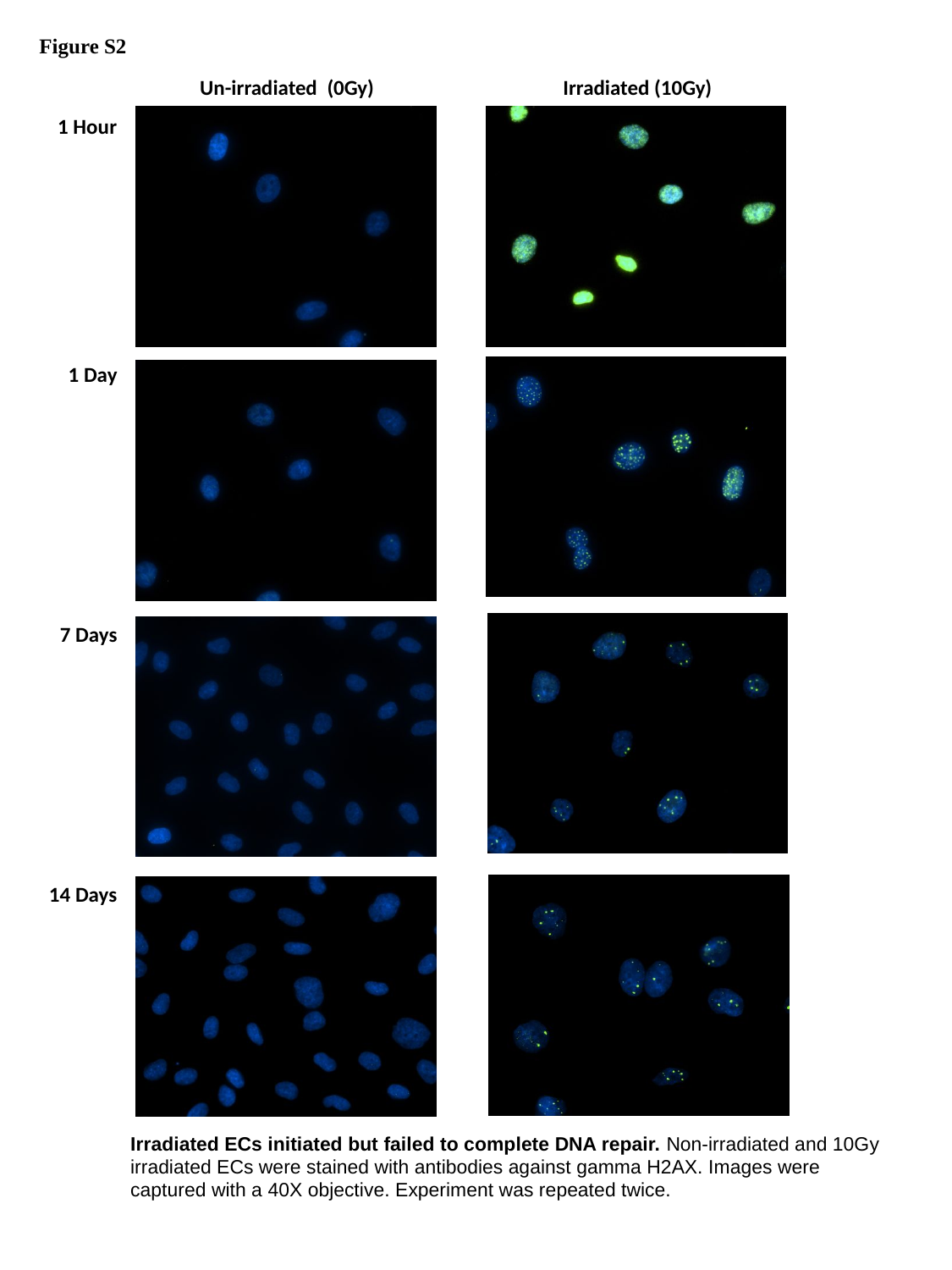

Figure S2
Un-irradiated (0Gy)
Irradiated (10Gy)
1 Hour
1 Day
7 Days
14 Days
Irradiated ECs initiated but failed to complete DNA repair. Non-irradiated and 10Gy
irradiated ECs were stained with antibodies against gamma H2AX. Images were
captured with a 40X objective. Experiment was repeated twice.

## Slide 3
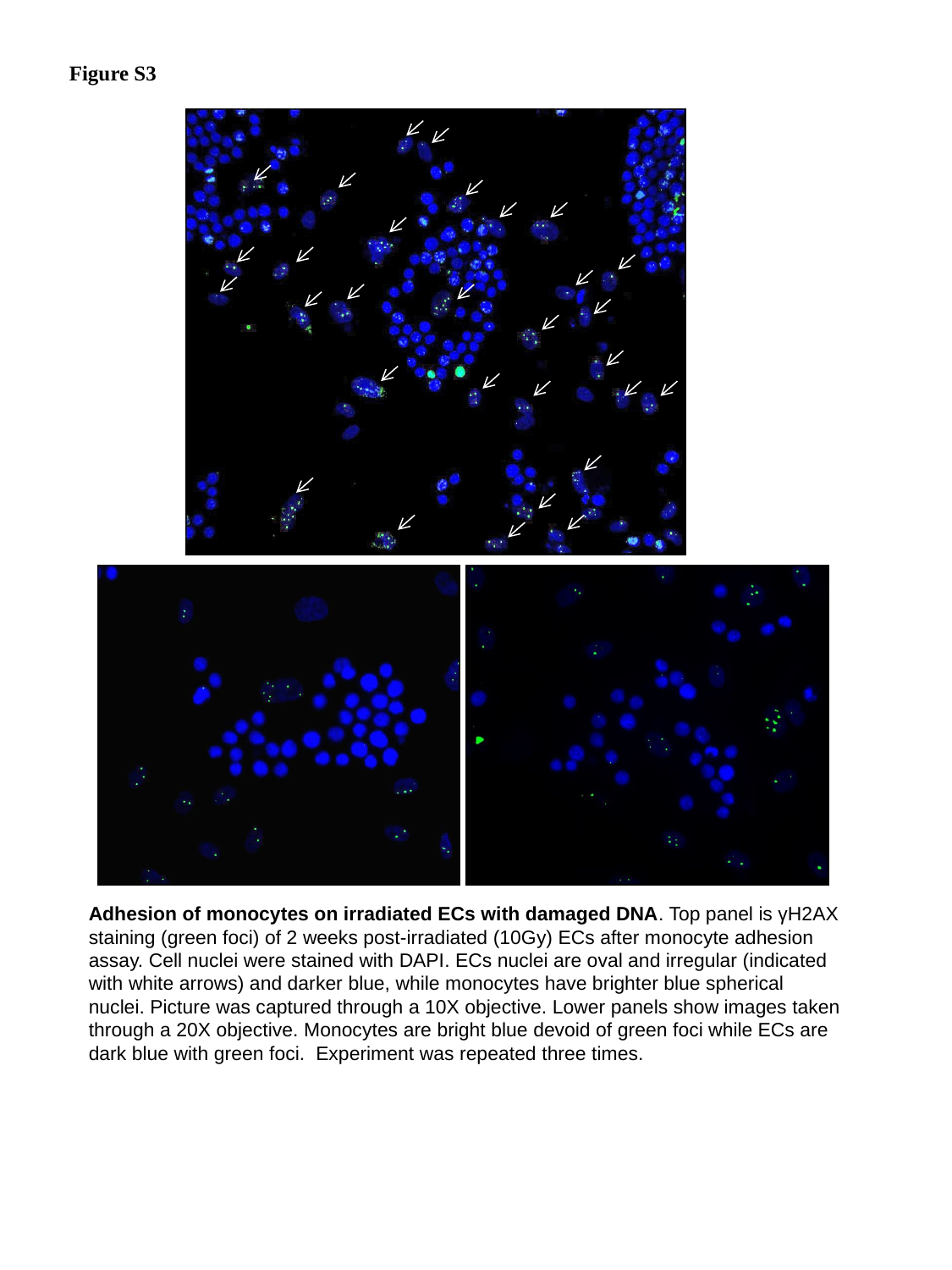

Figure S3
Adhesion of monocytes on irradiated ECs with damaged DNA. Top panel is γH2AX staining (green foci) of 2 weeks post-irradiated (10Gy) ECs after monocyte adhesion assay. Cell nuclei were stained with DAPI. ECs nuclei are oval and irregular (indicated with white arrows) and darker blue, while monocytes have brighter blue spherical nuclei. Picture was captured through a 10X objective. Lower panels show images taken through a 20X objective. Monocytes are bright blue devoid of green foci while ECs are dark blue with green foci. Experiment was repeated three times.

## Slide 4
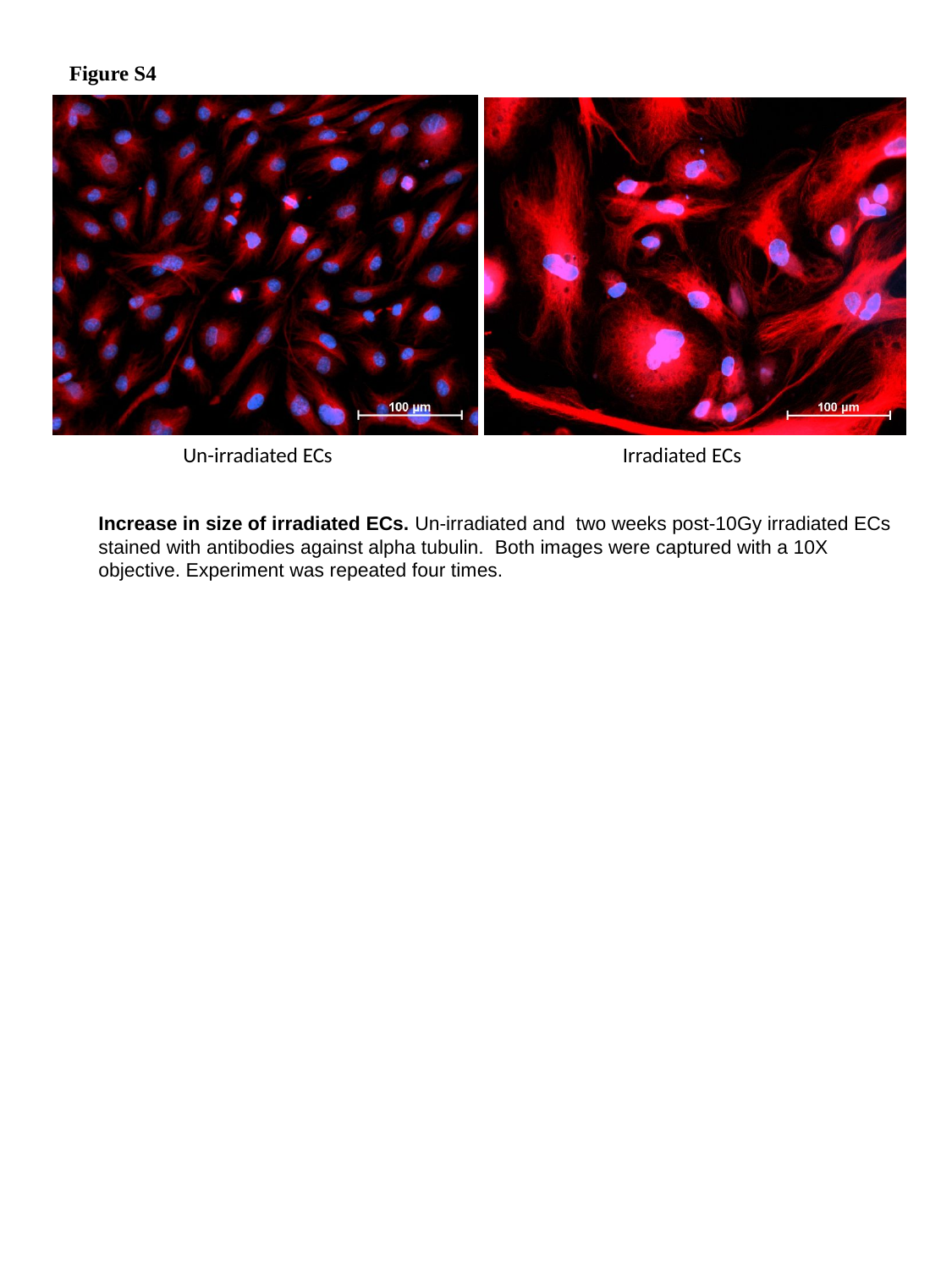

Figure S4
Un-irradiated ECs
Irradiated ECs
Increase in size of irradiated ECs. Un-irradiated and two weeks post-10Gy irradiated ECs stained with antibodies against alpha tubulin. Both images were captured with a 10X objective. Experiment was repeated four times.

## Slide 5
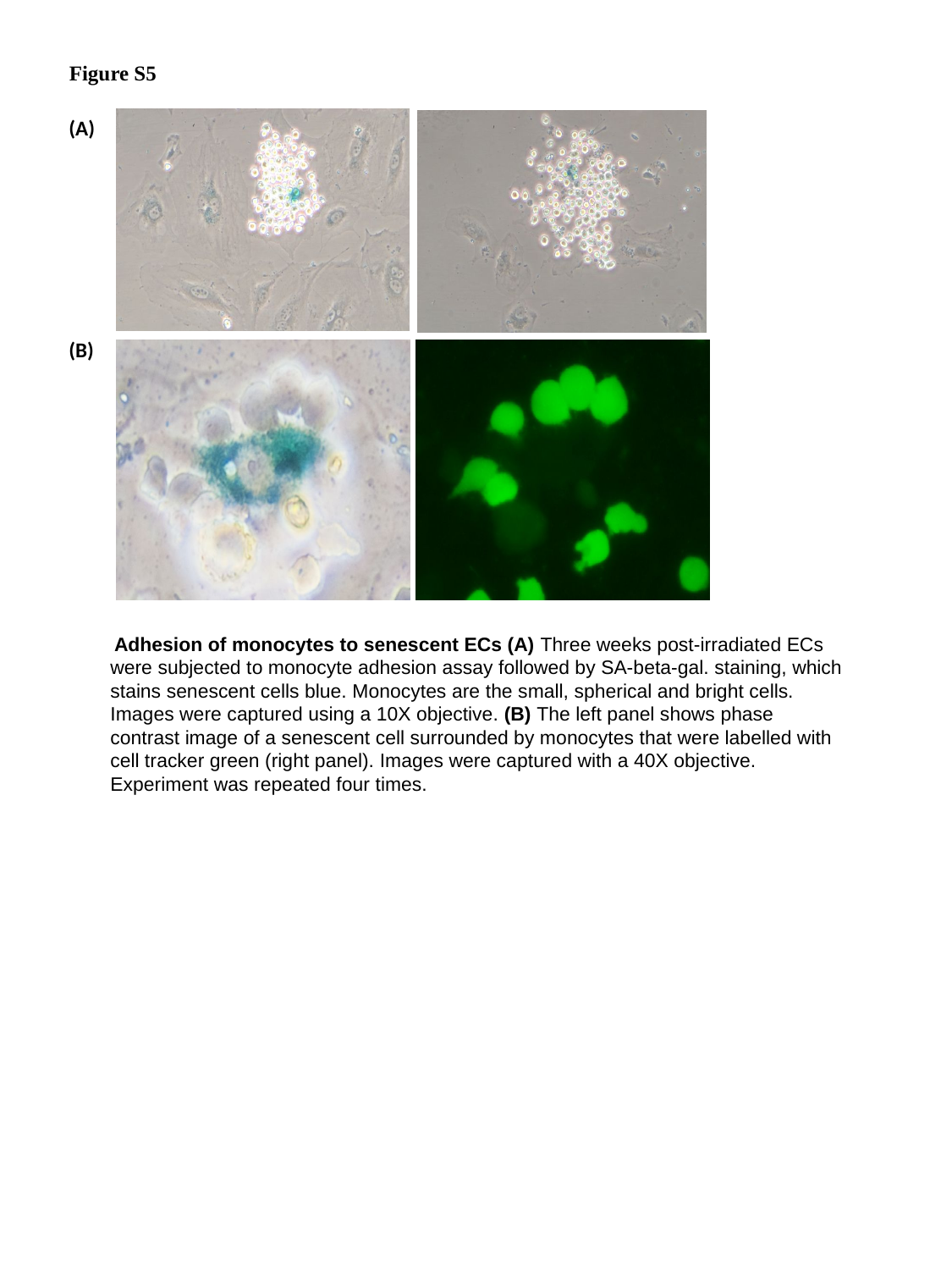

Figure S5
(A)
(B)
 Adhesion of monocytes to senescent ECs (A) Three weeks post-irradiated ECs were subjected to monocyte adhesion assay followed by SA-beta-gal. staining, which stains senescent cells blue. Monocytes are the small, spherical and bright cells. Images were captured using a 10X objective. (B) The left panel shows phase contrast image of a senescent cell surrounded by monocytes that were labelled with cell tracker green (right panel). Images were captured with a 40X objective. Experiment was repeated four times.

## Slide 6
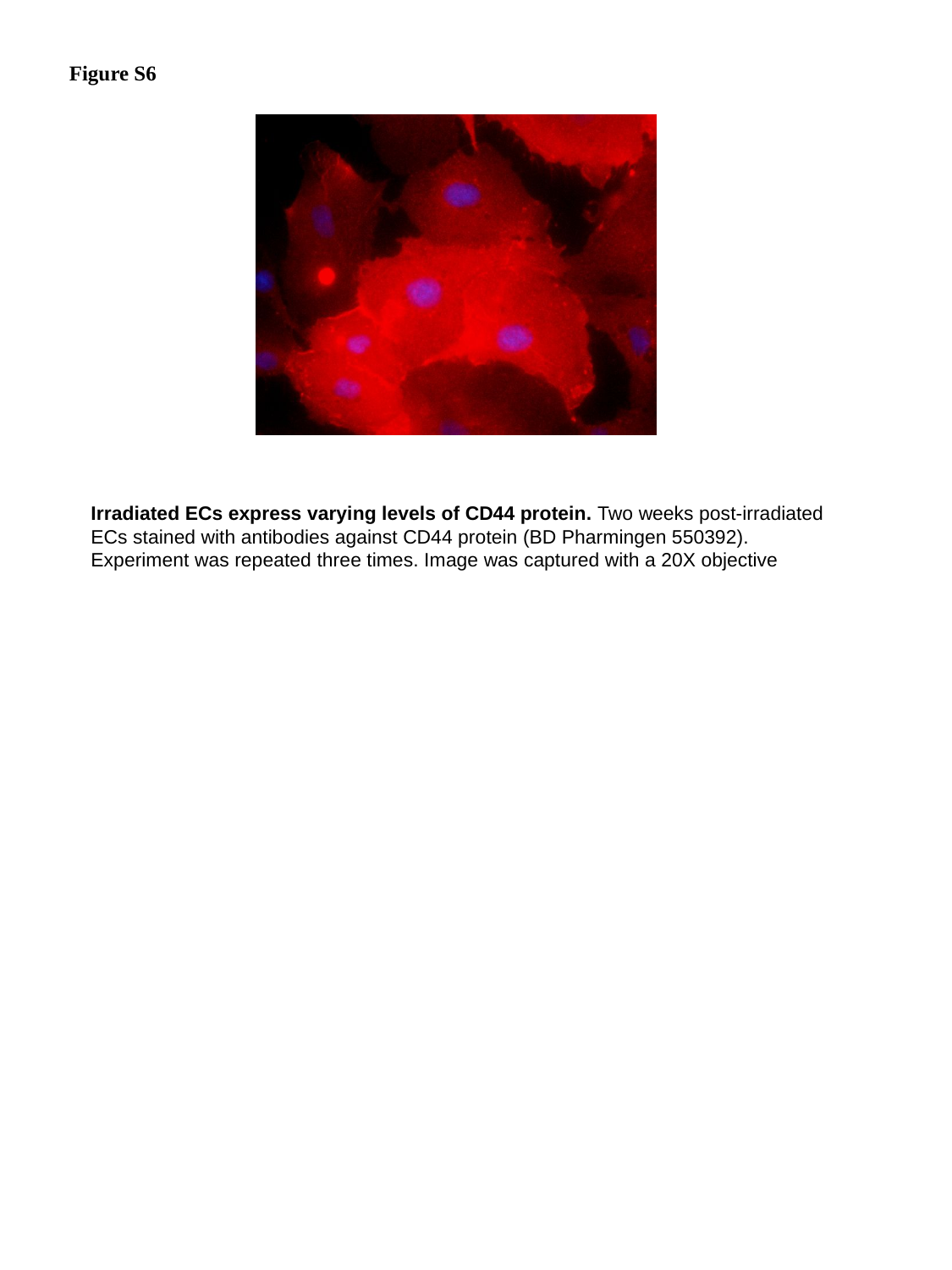

Figure S6
Irradiated ECs express varying levels of CD44 protein. Two weeks post-irradiated
ECs stained with antibodies against CD44 protein (BD Pharmingen 550392).
Experiment was repeated three times. Image was captured with a 20X objective

## Slide 7
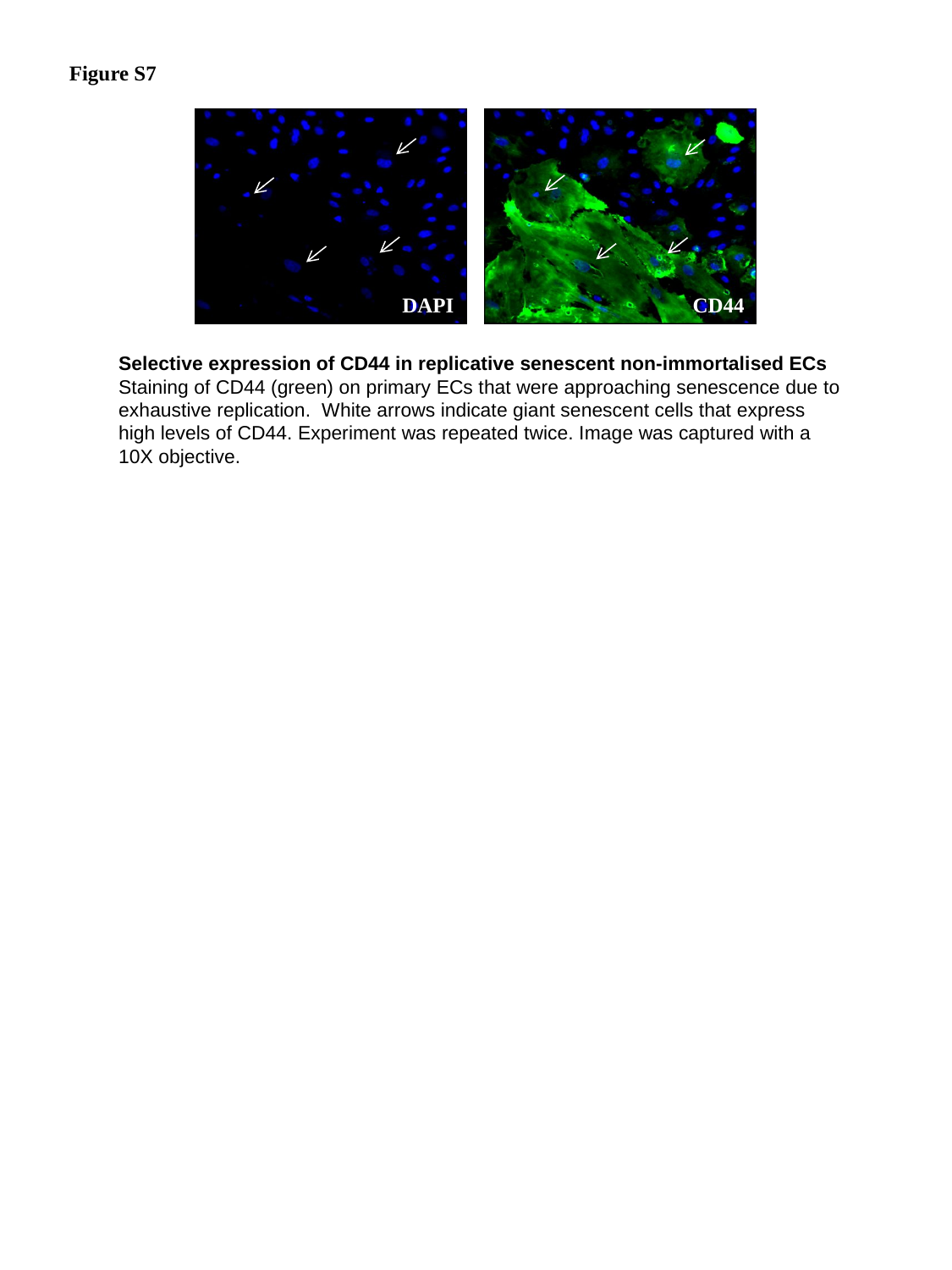

Figure S7
DAPI
CD44
Selective expression of CD44 in replicative senescent non-immortalised ECs Staining of CD44 (green) on primary ECs that were approaching senescence due to exhaustive replication. White arrows indicate giant senescent cells that express high levels of CD44. Experiment was repeated twice. Image was captured with a 10X objective.

## Slide 8
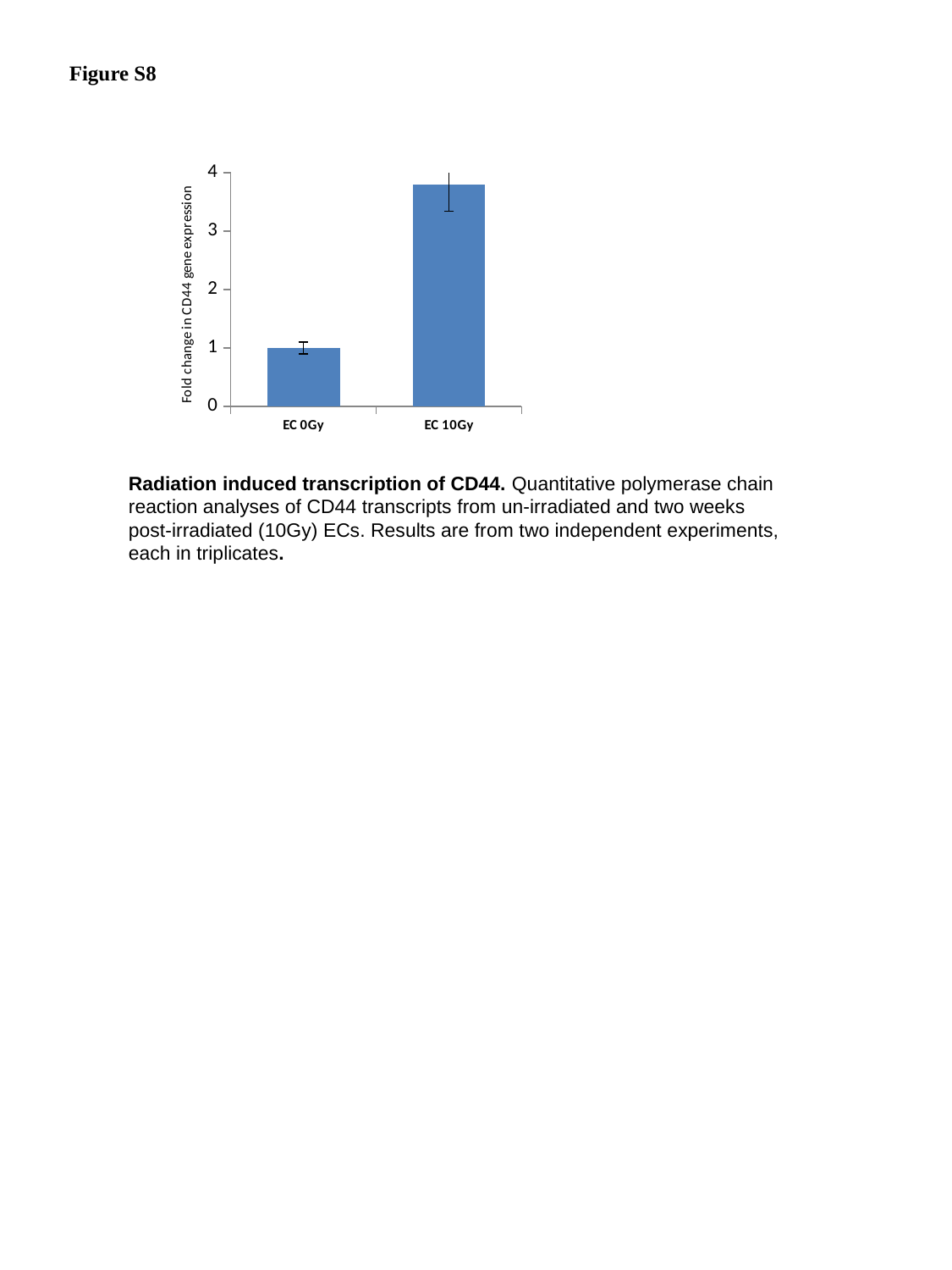

Figure S8
### Chart
| Category | Fold change |
|---|---|
| EC 0Gy | 1.0 |
| EC 10Gy | 3.7938591539234303 |Radiation induced transcription of CD44. Quantitative polymerase chain reaction analyses of CD44 transcripts from un-irradiated and two weeks post-irradiated (10Gy) ECs. Results are from two independent experiments, each in triplicates.

## Slide 9
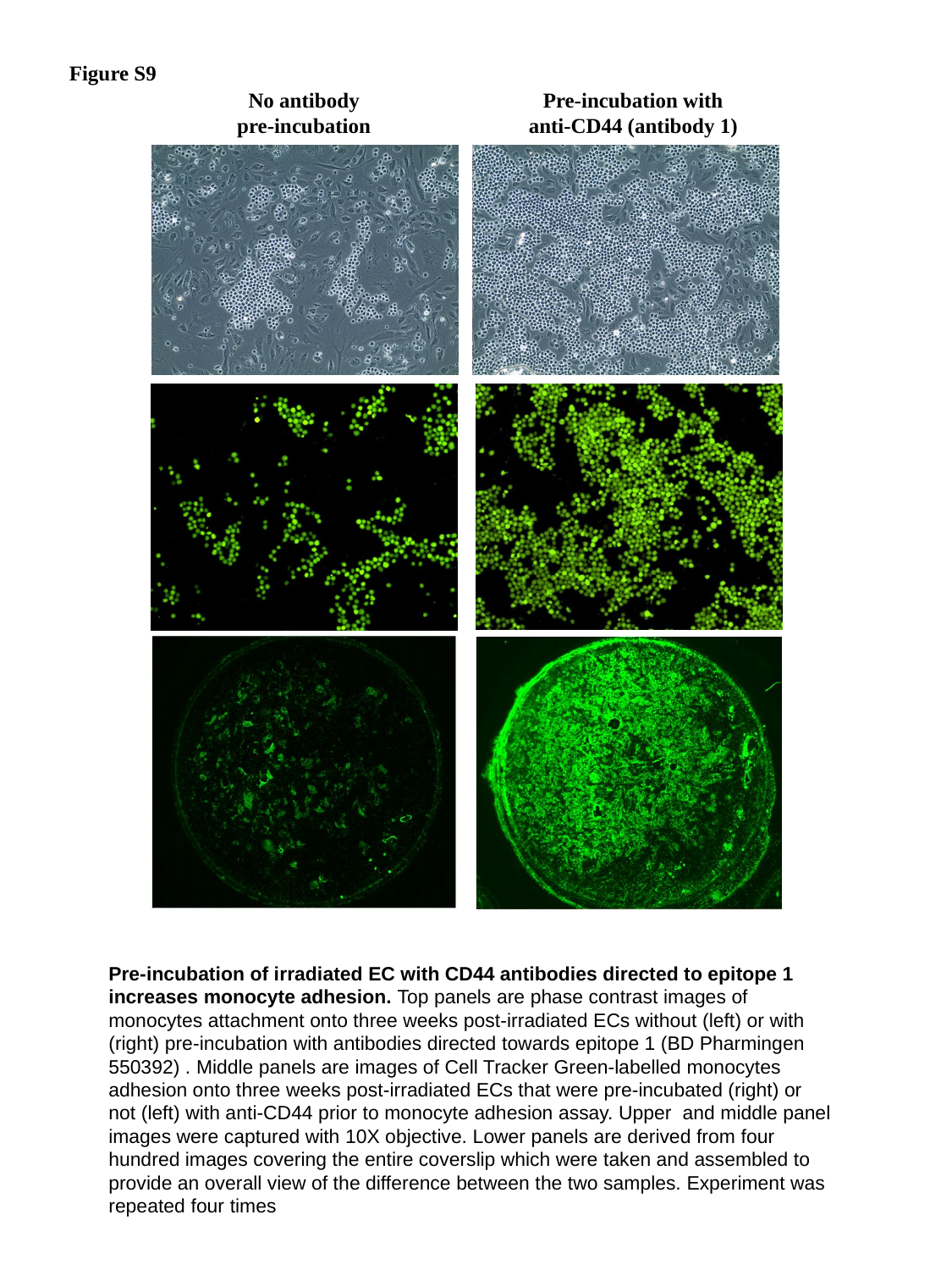

Figure S9
No antibody
pre-incubation
Pre-incubation with
anti-CD44 (antibody 1)
Pre-incubation of irradiated EC with CD44 antibodies directed to epitope 1 increases monocyte adhesion. Top panels are phase contrast images of monocytes attachment onto three weeks post-irradiated ECs without (left) or with (right) pre-incubation with antibodies directed towards epitope 1 (BD Pharmingen 550392) . Middle panels are images of Cell Tracker Green-labelled monocytes adhesion onto three weeks post-irradiated ECs that were pre-incubated (right) or not (left) with anti-CD44 prior to monocyte adhesion assay. Upper and middle panel images were captured with 10X objective. Lower panels are derived from four hundred images covering the entire coverslip which were taken and assembled to provide an overall view of the difference between the two samples. Experiment was repeated four times

## Slide 10
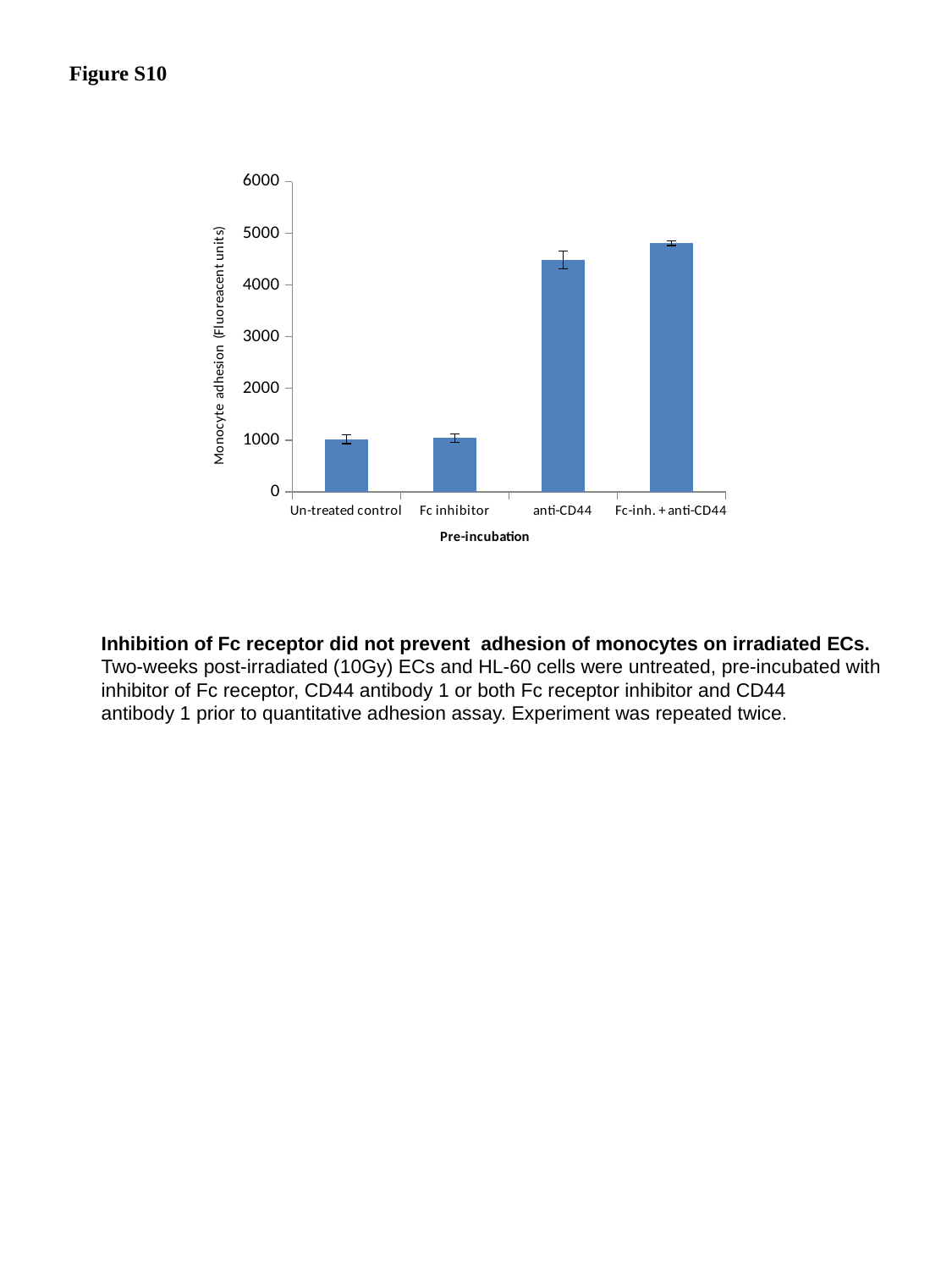

Figure S10
### Chart
| Category | |
|---|---|
| Un-treated control | 1014.0 |
| Fc inhibitor | 1036.3333333333292 |
| anti-CD44 | 4483.5 |
| Fc-inh. + anti-CD44 | 4809.0 |Inhibition of Fc receptor did not prevent adhesion of monocytes on irradiated ECs.
Two-weeks post-irradiated (10Gy) ECs and HL-60 cells were untreated, pre-incubated with
inhibitor of Fc receptor, CD44 antibody 1 or both Fc receptor inhibitor and CD44
antibody 1 prior to quantitative adhesion assay. Experiment was repeated twice.

## Slide 11
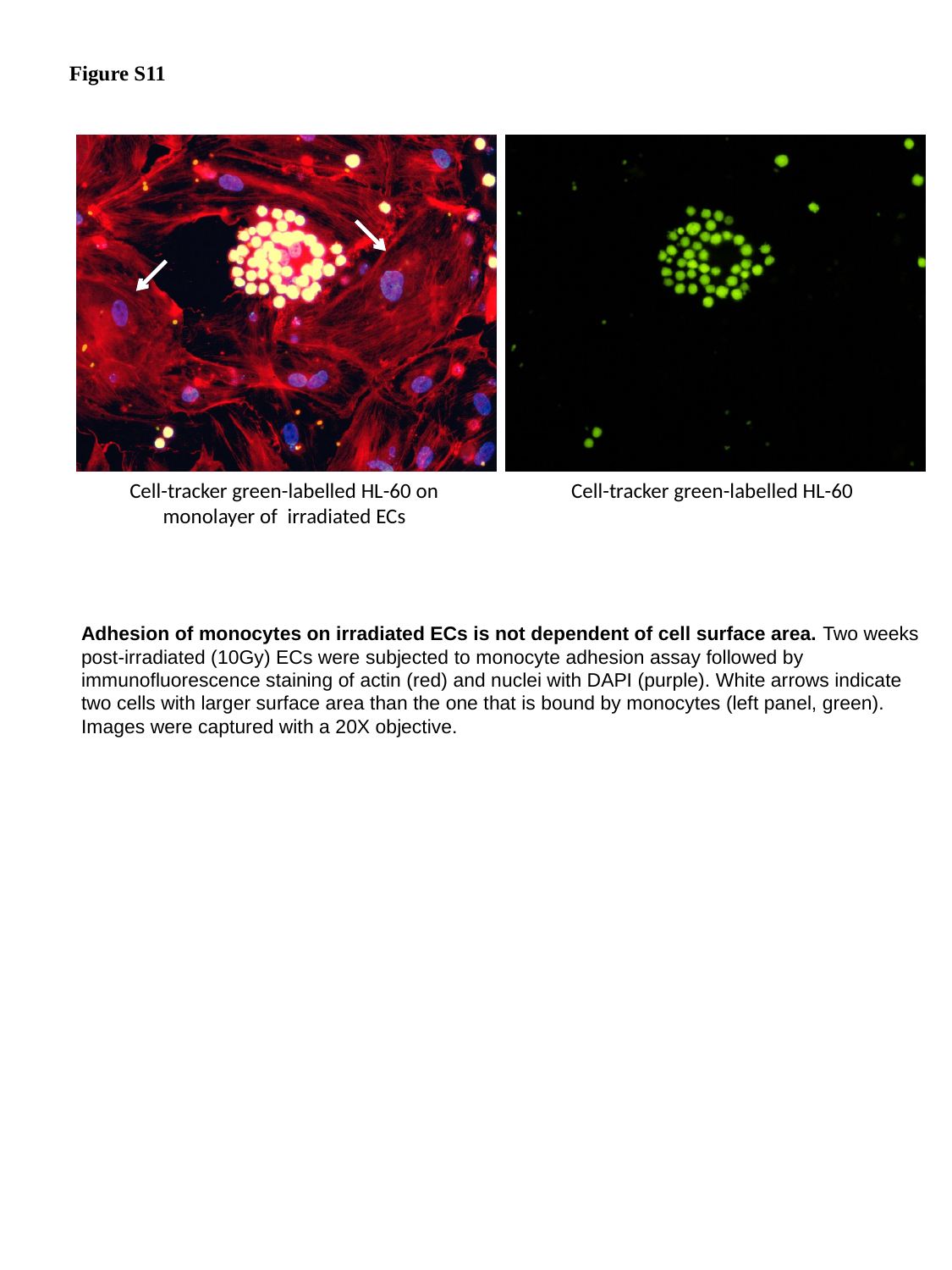

Figure S11
Cell-tracker green-labelled HL-60
Cell-tracker green-labelled HL-60 on
monolayer of irradiated ECs
Adhesion of monocytes on irradiated ECs is not dependent of cell surface area. Two weeks
post-irradiated (10Gy) ECs were subjected to monocyte adhesion assay followed by
immunofluorescence staining of actin (red) and nuclei with DAPI (purple). White arrows indicate
two cells with larger surface area than the one that is bound by monocytes (left panel, green).
Images were captured with a 20X objective.

## Slide 12
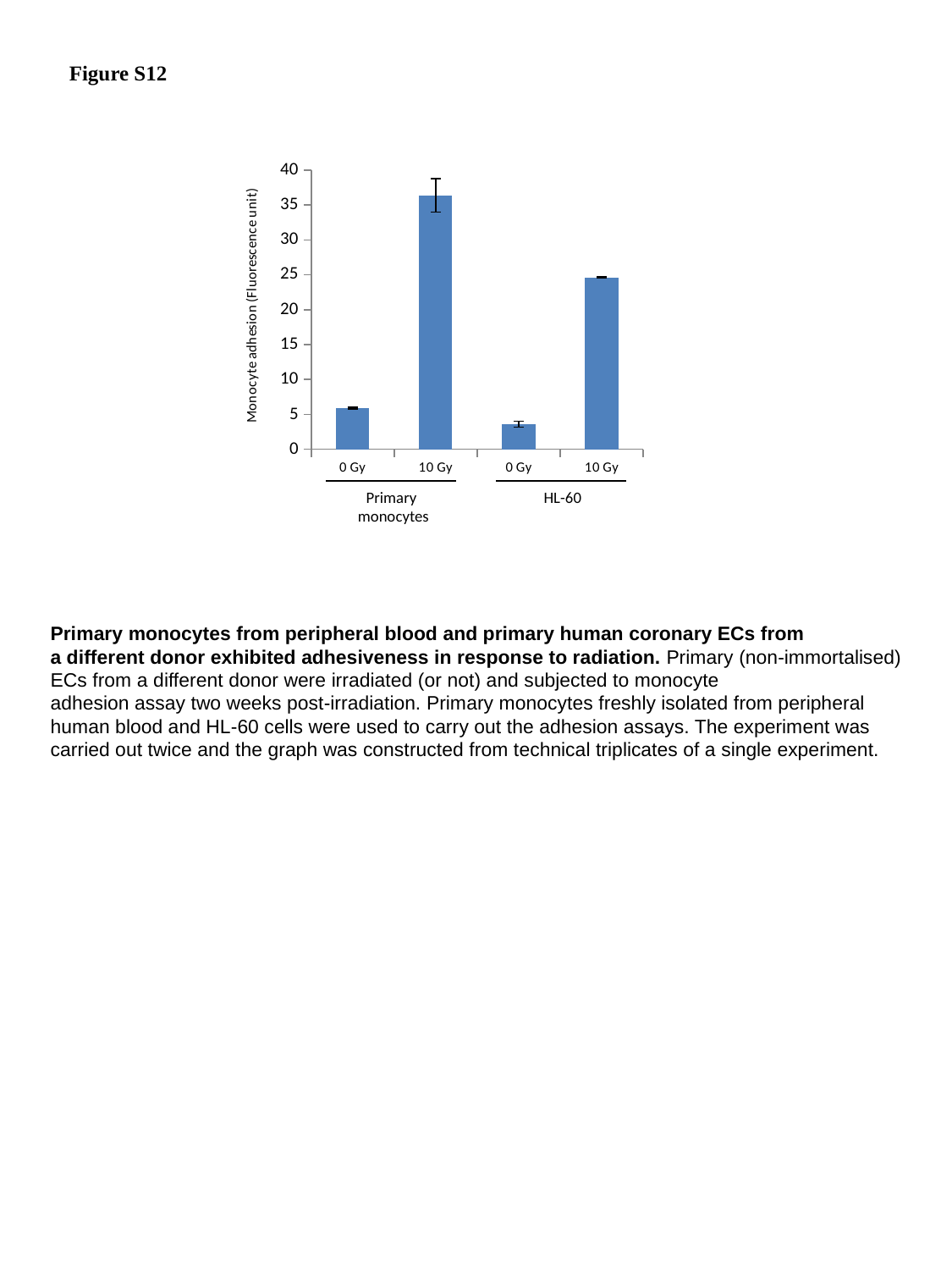

Figure S12
### Chart
| Category | |
|---|---|
| 0 Gy | 5.9 |
| 10 Gy | 36.4 |
| 0 Gy | 3.6 |
| 10 Gy | 24.6 |Primary
monocytes
HL-60
Primary monocytes from peripheral blood and primary human coronary ECs from
a different donor exhibited adhesiveness in response to radiation. Primary (non-immortalised)
ECs from a different donor were irradiated (or not) and subjected to monocyte
adhesion assay two weeks post-irradiation. Primary monocytes freshly isolated from peripheral
human blood and HL-60 cells were used to carry out the adhesion assays. The experiment was
carried out twice and the graph was constructed from technical triplicates of a single experiment.
